# Supplementary material for: Functional transcription factor target discovery via compendia of binding and expression profiles
Source: Sci Rep. 2016 Feb 9;6:20649. doi: 10.1038/srep20649 (PMC4746627; doi:10.1038/srep20649)
Supplement: Supplementary Information [file srep20649-s1.pdf]

Functional transcription factor target discovery via compendia of binding and expression profiles – Supplementary Material

Christopher J. Banks, Anagha Joshi, and Tom Michoel

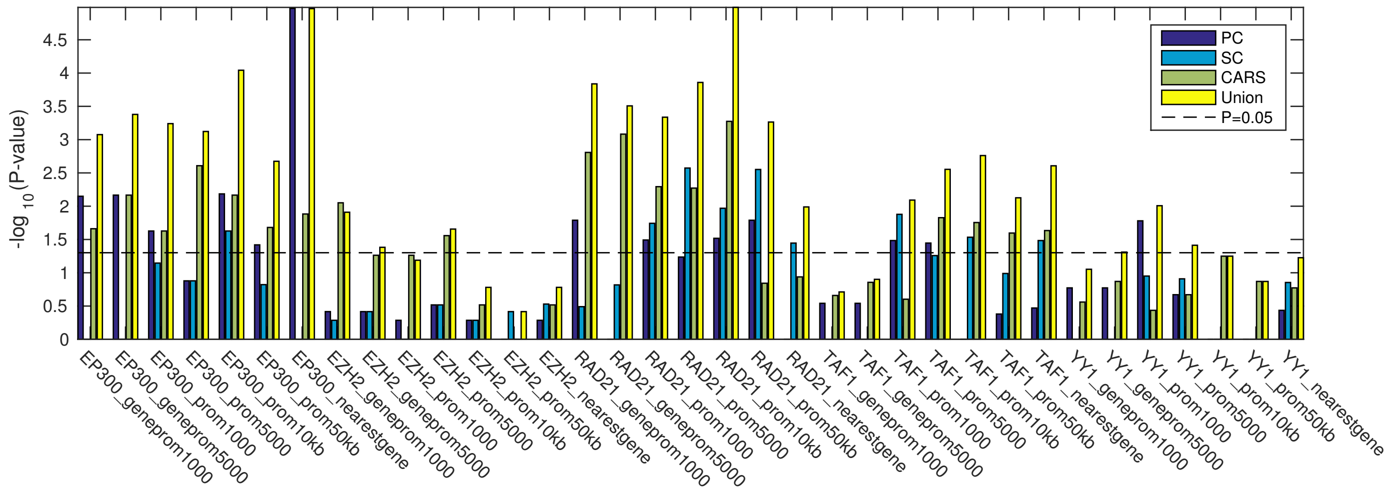

Figure 1: Functional target set significance (hypergeometric P-value) predicted by each of the correlation methods for all peak-to-gene models at a predicted 1.5-fold precision over background.

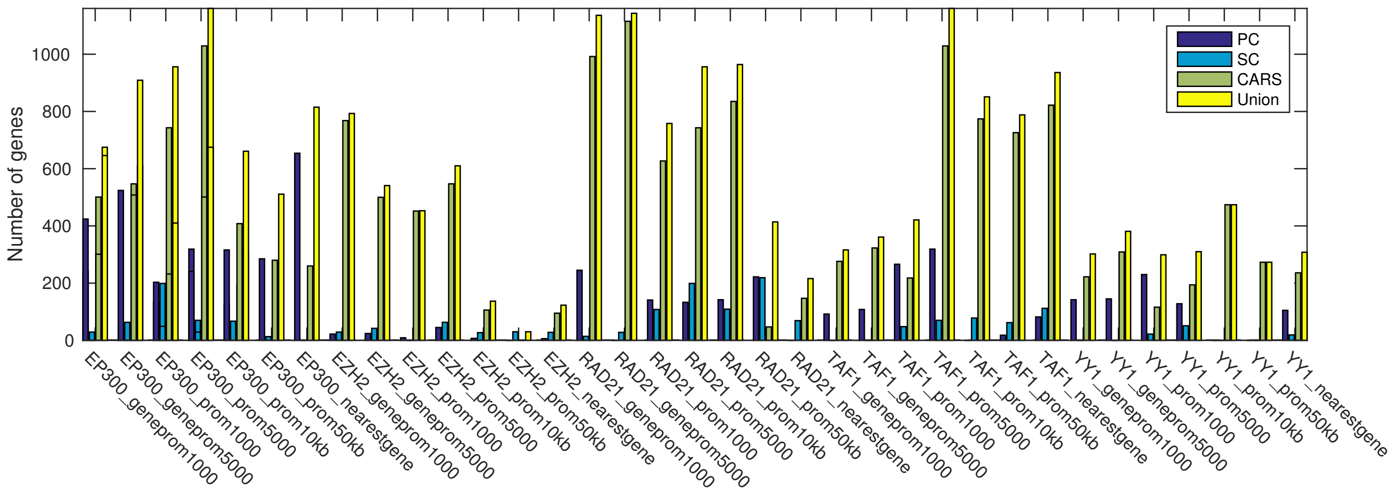

Figure 2: Functional target set sizes predicted by each of the correlation methods for all peak-to-gene models at a predicted 1.5-fold precision over background. Set sizes refer to subsets of the complete set of 24,392 genes exceeding the 1.5-fold precision threshold determined by analysing the 8,872 reference genes.

TF	Model	Binding			MB	
		Background	Bound	p	n	p
EP300	geneprom1000	0.385	0.424	**0.000858**	0	1
	geneprom5000	0.385	0.425	**0.00048**	0	1
	prom1000	0.385	0.394	0.347	0	1
	prom5000	0.385	0.413	0.0512	0	1
	prom10kb	0.385	0.426	**0.00679**	23	**0.0243**
	prom50kb	0.385	0.445	**2.97e-06**	86	**0.000156**
	nearestgene	0.385	0.447	**1.03e-07**	29	**0.0218**
EZH2	geneprom1000	0.216	0.186	0.923	0	1
	geneprom5000	0.216	0.197	0.827	0	1
	prom1000	0.216	0.178	0.877	0	1
	prom5000	0.216	0.216	0.529	0	1
	prom10kb	0.216	0.21	0.607	0	1
	prom50kb	0.216	0.212	0.59	0	1
	nearestgene	0.216	0.205	0.72	0	1
RAD21	geneprom1000	0.38	0.398	**0.000132**	18	**0.0392**
	geneprom5000	0.38	0.4	**5.55e-06**	19	0.0618
	prom1000	0.38	0.391	0.173	6	0.152
	prom5000	0.38	0.415	**3.7e-06**	12	0.125
	prom10kb	0.38	0.423	**6.05e-10**	47	**0.00509**
	prom50kb	0.38	0.405	**3.08e-08**	0	1
	nearestgene	0.38	0.402	**9.34e-08**	1	0.38
TAF1	geneprom1000	0.236	0.229	0.995	4	0.239
	geneprom5000	0.236	0.229	0.995	4	0.239
	prom1000	0.236	0.23	0.938	0	1
	prom5000	0.236	0.231	0.947	13	0.173
	prom10kb	0.236	0.229	0.977	0	1
	prom50kb	0.236	0.23	0.953	4	0.239
	nearestgene	0.236	0.232	0.924	4	0.239
YY1	geneprom1000	0.311	0.308	0.835	0	1
	geneprom5000	0.311	0.307	0.893	0	1
	prom1000	0.311	0.305	0.919	0	1
	prom5000	0.311	0.309	0.712	5	**0.0349**
	prom10kb	0.311	0.31	0.57	14	0.109
	prom50kb	0.311	0.308	0.756	3	0.229
	nearestgene	0.311	0.308	0.791	4	0.367

Table 1: Overlap between various predicted and known functional TF-target sets for ENCODE data. Binding: the ratio of differentially expressed genes among all 8,872 reference genes (Background) and among genes bound by the TF in the given peak-to-gene model (Bound), and the hypergeometric overlap P-value (p). Multiple Bind: gene sets predicted by a threshold on the number of peaks with a 1.5-fold increase in ratio of differentially expressed genes compared to the background, showing the number of genes (n) and hypergeometric overlap P-value (p). All subset sizes refer to the number of 8,872 reference genes exceeding the threshold. Significant P-values (< 0.05) are indicated in bold

TF	Model	PC		SC		CARS		Union	
		n	p	n	p	n	p	n	p
EP300	geneprom1000	41	**0.00709**	0	1	29	**0.0218**	63	**0.00084**
	geneprom5000	43	**0.00681**	0	1	43	**0.00681**	78	**0.000418**
	prom1000	25	**0.0236**	17	0.0715	25	**0.0236**	58	**0.000575**
	prom5000	12	0.132	12	0.132	51	**0.00246**	69	**0.000754**
	prom10kb	45	**0.00653**	25	**0.0236**	43	**0.00681**	93	**9.07e-05**
	prom50kb	24	**0.0381**	8	0.151	31	**0.0209**	59	**0.00211**
	nearestgene	119	**1.07e-05**	0	1	32	**0.0131**	138	**1.08e-05**
EZH2	geneprom1000	6	0.383	3	0.518	92	**0.00889**	94	**0.0123**
	geneprom5000	6	0.383	6	0.383	46	0.0546	52	**0.0415**
	prom1000	3	0.518	0	1	46	0.0546	47	0.0648
	prom5000	9	0.304	9	0.304	61	**0.0277**	71	**0.0221**
	prom10kb	3	0.518	3	0.518	9	0.304	14	0.166
	prom50kb	0	1	6	0.383	0	1	6	0.383
	nearestgene	3	0.518	5	0.295	9	0.304	14	0.166
RAD21	geneprom1000	35	**0.0162**	3	0.323	61	**0.00156**	91	**0.000145**
	geneprom5000	0	1	6	0.152	70	**0.000826**	76	**0.000311**
	prom1000	26	**0.0322**	31	**0.0181**	47	**0.00509**	92	**0.00046**
	prom5000	21	0.058	56	**0.00267**	45	**0.00534**	108	**0.000138**
	prom10kb	28	**0.0304**	34	**0.0107**	71	**0.000532**	116	**1.03e-05**
	prom50kb	35	**0.0162**	54	**0.00281**	8	0.144	86	**0.000544**
	nearestgene	0	1	22	**0.0358**	14	0.115	36	**0.0103**
TAF1	geneprom1000	8	0.288	0	1	14	0.219	17	0.194
	geneprom5000	8	0.288	0	1	19	0.139	22	0.126
	prom1000	50	**0.0328**	18	**0.0133**	11	0.25	67	**0.00809**
	prom5000	47	**0.0358**	32	0.0552	70	**0.0149**	130	**0.0028**
	prom10kb	0	1	36	**0.0293**	64	**0.0176**	100	**0.00173**
	prom50kb	2	0.417	28	0.103	59	**0.0253**	87	**0.00748**
	nearestgene	5	0.338	50	**0.0328**	62	**0.0232**	112	**0.00247**
YY1	geneprom1000	10	0.169	0	1	6	0.275	16	**0.0886**
	geneprom5000	10	0.169	0	1	12	0.135	22	**0.0489**
	prom1000	47	**0.0166**	9	0.112	4	0.367	53	**0.00983**
	prom5000	8	0.213	17	0.123	8	0.213	29	**0.0386**
	prom10kb	0	1	0	1	25	0.0563	25	0.0563
	prom50kb	0	1	0	1	12	0.135	12	0.135
	nearestgene	4	0.367	7	0.14	10	0.169	20	0.0594

Table 2: Overlap between various predicted and known functional TF-target sets for ENCODE data. Pearson Correlation (PC), Spearman Correlation (SC), Combined Angle Ratio Statistic (CARS), and Union: gene sets predicted by a threshold on the correlation score, for each method respectively and for the union of those sets, with a 1.5-fold increase in ratio of differentially expressed genes compared to the background, showing the number of genes (n) and hypergeometric overlap P-value (p). All subset sizes refer to the number of 8,872 reference genes exceeding the threshold. Significant P-values (< 0.05) are indicated in bold

		Binding			MB	
TF	Model	Background	Bound	p	n	p
Per2	geneprom1kb	0.281	0.281	0.486	1	0.281
	geneprom5kb	0.281	0.282	0.266	11	0.17
	prom1kb	0.268	0.269	0.397	0	1
	prom5kb	0.272	0.277	**0.0414**	0	1
	prom10kb	0.28	0.284	0.0535	30	**0.0217**
	prom50kb	0.279	0.281	0.141	21	0.102
	nearestgene	0.278	0.28	0.101	2	0.478
Cry1	geneprom1kb	0.0126	0.0126	0.734	939	**0.0196**
	geneprom5kb	0.0129	0.0129	0.726	580	0.0652
	prom1kb	0.0109	0.0108	0.796	70	**0.0393**
	prom5kb	0.0123	0.0124	0.644	365	0.0752
	prom10kb	0.0124	0.0125	0.562	234	0.068
	prom50kb	0.0127	0.0128	0.439	213	0.132
	nearestgene	0.0127	0.0127	0.7	916	**0.035**
Cry2	geneprom1kb	0.014	0.0136	0.897	1040	**0.0177**
	geneprom5kb	0.014	0.0136	0.896	185	0.257
	prom1kb	0.0128	0.0121	0.903	171	0.171
	prom5kb	0.013	0.0125	0.89	106	0.157
	prom10kb	0.014	0.0134	0.932	24	0.287
	prom50kb	0.0149	0.0146	0.858	103	0.198
	nearestgene	0.0148	0.0147	0.701	378	0.103

Table 3: Overlap between various predicted and known functional TF-target sets for mouse circadian data. Binding: the ratio of differentially expressed genes among all 8,872 reference genes (Background) and among genes bound by the TF in the given peak-to-gene model (Bound), and the hypergeometric overlap P-value (p). Multiple Bind: gene sets predicted by a threshold on the number of peaks with a 1.5-fold increase in ratio of differentially expressed genes compared to the background, showing the number of genes (n) and hypergeometric overlap P-value (p). Significant P-values (< 0.05) are indicated in bold

		PC		SC		CARS		Union	
TF	Model	n	p	n	p	n	p	n	p
Per2	geneprom1kb	2	0.483	10	0.119	0	1	10	0.119
	geneprom5kb	0	1	10	0.119	0	1	10	0.119
	prom1kb	4	0.292	2	0.464	0	1	6	0.198
	prom5kb	17	0.153	3	0.182	0	1	19	0.117
	prom10kb	0	1	9	0.225	0	1	9	0.225
	prom50kb	0	1	0	1	0	1	0	1
	nearestgene	0	1	0	1	0	1	0	1
Cry1	geneprom1kb	2164	**0.00039**	1728	**0.000496**	528	0.124	2432	**0.000524**
	geneprom5kb	1807	**0.00239**	1807	**0.00239**	619	0.0959	2288	**0.000677**
	prom1kb	856	0.0549	61	0.491	367	0.203	1015	0.0509
	prom5kb	1026	**0.0301**	702	0.0778	486	0.137	1298	**0.0117**
	prom10kb	268	0.238	45	0.107	536	0.121	691	0.138
	prom50kb	420	0.16	1204	**0.0207**	473	0.142	1493	**0.012**
	nearestgene	787	**0.0671**	1393	**0.0101**	577	0.111	1760	**0.00607**
Cry2	geneprom1kb	1570	**0.00144**	566	0.087	54	0.536	1609	**0.00238**
	geneprom5kb	1670	**0.00103**	608	**0.0356**	95	0.385	1727	**0.000851**
	prom1kb	622	0.0659	229	0.162	0	1	622	0.0659
	prom5kb	974	**0.0201**	422	**0.0368**	512	0.111	1144	**0.00134**
	prom10kb	1145	**0.0095**	427	**0.0287**	572	0.0873	1339	**0.00123**
	prom50kb	44	0.485	0	1	760	**0.0484**	773	0.0555
	nearestgene	1352	**0.00667**	774	**0.0289**	135	0.322	1468	**0.00298**

Table 4: Overlap between various predicted and known functional TF-target sets for mouse circadian data. Pearson Correlation (PC), Spearman Correlation (SC), Combined Angle Ratio Statistic (CARS), and Union: gene sets predicted by a threshold on the correlation score, for each method respectively and for the union of those sets, with a 1.5-fold increase in ratio of differentially expressed genes compared to the background, showing the number of genes (n) and hypergeometric overlap P-value (p). Significant P-values (< 0.05) are indicated in bold

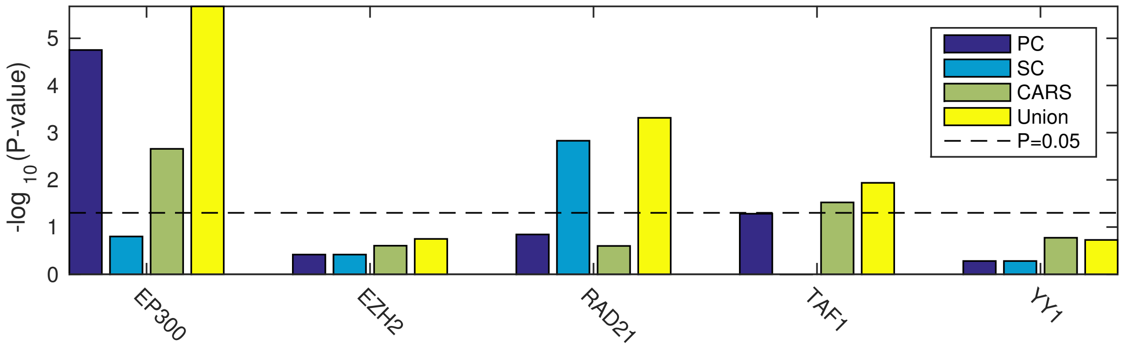

Figure 3: Functional target set significance (hypergeometric P-value) using quantitative (sum of peak heights) ChIP-seq data predicted by each of the correlation methods for the prom5kb peak-to-gene model at a predicted 1.5-fold precision over background.

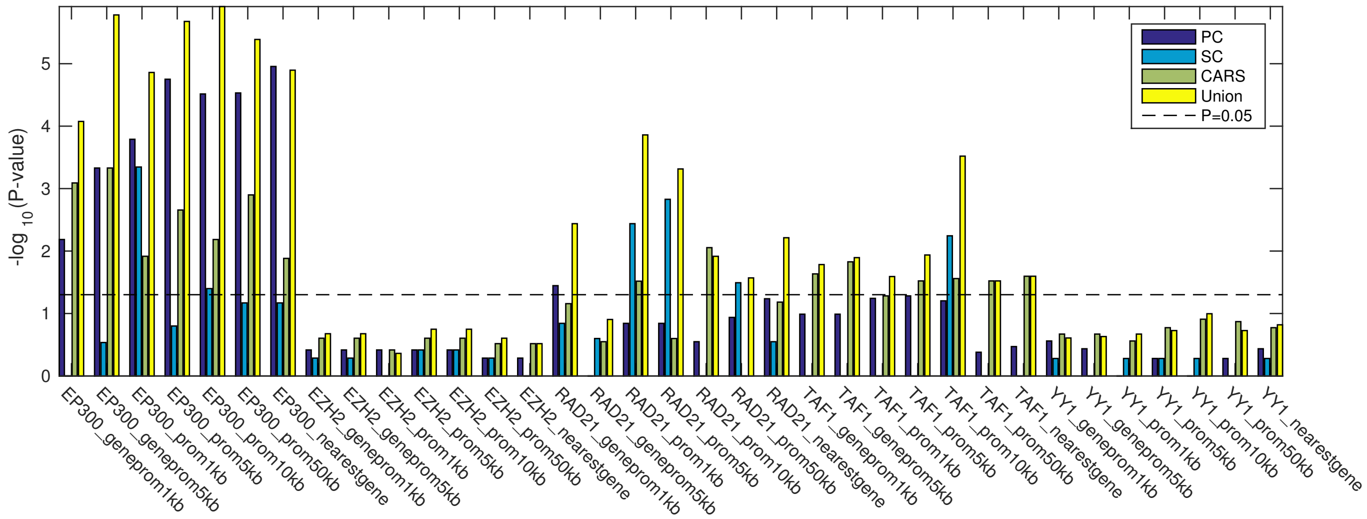

Figure 4: Functional target set significance (hypergeometric P-value) using quantitative (sum of peak heights) ChIP-seq data predicted by each of the correlation methods for all peak-to-gene models at a predicted 1.5-fold precision over background.

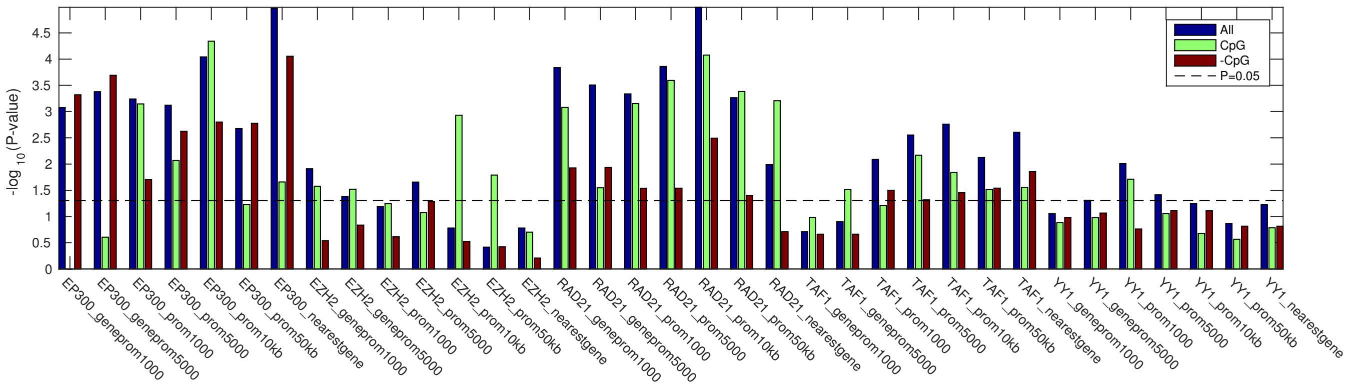

Figure 5: Functional target set significance (hypergeometric P-value), using CpG partitioned datasets, predicted by each of the correlation methods for all peak-to-gene models at a predicted 1.5-fold precision over background. We show significance using all genes (blue), only CpG-rich promoters (green), and only CpG-depleted promoters (red).

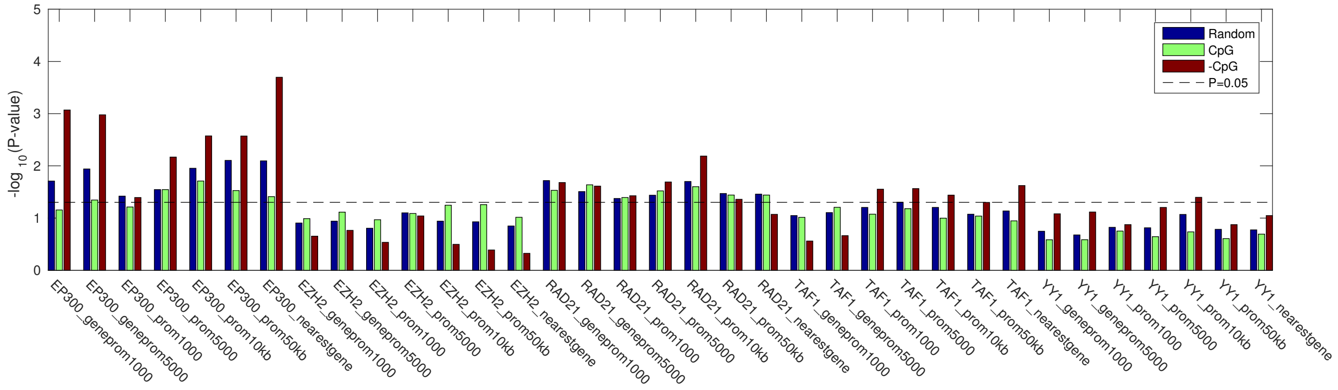

Figure 6: Functional target set significance (hypergeometric P-value), using CpG partitioned datasets, each of 1000 randomly selected genes, predicted by each of the correlation methods for all peak-to-gene models at a predicted 1.5-fold precision over background. We show significance using all genes (blue), only CpG-rich promoters (green), and only CpG-depleted promoters (red). All results are the average of 100 random samples of 1000 genes per set.

Data	TFs	Source
Human		
ChIP-seq	EP300<br>EZH2<br>RAD21<br>TAF1<br>YY1<br>CEBPB<br>MYC<br>REST	https://genome.ucsc.edu/ENCODE/dataMatrix/encodeChipMatrixHuman.html
RNA-seq	EP300<br>EZH2<br>RAD21<br>TAF1<br>YY1<br>CEBPB<br>MYC<br>REST	https://genome.ucsc.edu/ENCODE/dataMatrix/encodeDataMatrixHuman.html
Knockout	EP300<br>EZH2<br>RAD21<br>TAF1<br>YY1	Cusanovich et al. (2014)
Mouse		
ChIP-seq<br>&<br>RNA-seq	Bmal1<br>Clock<br>Cry1<br>Cry2<br>Per1<br>Per2	Koike et al. (2012)
Knockout	Per2	http://www.ncbi.nlm.nih.gov/geo/query/acc.cgi?acc=GSE30139

Table 5: Data sources.
