# Supplementary material for: Functional transcription factor target discovery via compendia of binding and expression profiles
Source: Sci Rep. 2016 Feb 9;6:20649. doi: 10.1038/srep20649 (PMC4746627; doi:10.1038/srep20649)
Supplement: Supplementary Dataset [file srep20649-s2.zip › Supplementary Dataset/mtsdown.htm]

Nature


|  |
| --- |
|  |

|  |
| --- |
| The site you are trying to access is currently unavailable. Access to the site should be restored shortly. Please try again later.  If the site continues to be unavailable, or you keep being redirected to this page, please contact us at the Platform Support service page.  We apologize for any inconvenience this may cause. |
|  |

|  |  |
| --- | --- |
| ........................................................................................................................................................................................... | |
|  |  |
